# Characteristics of cracks in posterior teeth and factors associated with symptoms: a cross‐sectional practice‐based observational study

**DOI:** 10.1111/adj.13075

**Published:** 2025-04-24

**Authors:** P Renner, U Krishnan, A Moule, M Swain

**Affiliations:** ^1^ School of Dentistry The University of Queensland Brisbane Queensland Australia; ^2^ School of Aerospace, Mechanical and Mechatronics Engineering University of Sydney Sydney New South Wales Australia

**Keywords:** Cracked teeth, cuspal cracks, cold sensitivity, dentine hypersensitivity, biting pain

## Abstract

**Background:**

Cracks in teeth are the third most common reason for tooth loss. The primary aim of this study was to identify the patient‐specific and tooth‐specific characteristics of individuals who presented to a private general dental practice with cracked posterior teeth. The secondary aim was to explore the relationship between the above characteristics and investigate their influence on signs and symptoms.

**Materials and Methods:**

A total of 147 records were analysed in terms of patient‐specific, tooth‐specific and crack‐specific characteristics. Initial data analysis involved the use of descriptive statistics and chi‐square tests. Stepwise logistic regression was used for model building, and further data analysis was performed using binomial and multinomial logistic regression.

**Results:**

Most cracked teeth were asymptomatic (55.1%) and were not visible before removal of restoration. Unrestored marginal ridges (OR2.89), cracks visible before restoration removal (OR3.04) and cracks involving both the body and cusps of teeth (OR3.11) were associated with cold sensitivity. Not all cracked teeth were positive for the bite test. Molar teeth (OR8.79) and those with amalgam restoration (OR4.81) were associated with intersecting cracks.

**Conclusion:**

The presentation of teeth with cracks in general dental practice seems to differ from that reported in the literature from specialist practice.

**Abbreviations and acronyms**
AIC: Akaike information criterion
LTMs: low threshold mechanoreceptors
NDPBRN: national dental practice‐based research network
VIF: variance inflation factor

## INTRODUCTION

Cracks in teeth are the third most common reason for tooth loss in industrialised countries.^1^ The global prevalence of cracked teeth is difficult to estimate, with reports ranging from around 10%^2^ in 2007 to 70%^3^ in 2011. The incidence of cracked teeth also varies according to tooth type, with mandibular molars being involved in 28%–70% compared with maxillary molars reported in 19%–57% of studies.^4^ Dentists estimate they treat around 3.2 cracked teeth per week,^5^, ^6^ with a recent increase reported during the period of the Covid pandemic.^7^

The identification of cracks in teeth and discerning the ones that require intervention can be difficult, due principally to three different factors. These include the variability of symptoms, poor localisation of the pain, symptoms mistaken as dentine hypersensitivity and inconsistent results of diagnostic tests.^8^ The variability of presenting symptoms is the key factor, with the literature skewed to presentations in specialist endodontic practices.^2^, ^9^, ^10^ Cracked teeth symptoms can range from being asymptomatic to having cold sensitivity, pain on biting, sensitivity to heat, spontaneous pain and long‐term constant aches.^4^ The inability to localise the pain, particularly in an acute presentation, due to poor two‐point discrimination and pain referral, often associated with central sensitisation, is one of the challenges.^11^ Finally, the diagnostic testing can yield false positive results.^8^ However, it is important to realise that cracks in teeth are a physical entity, and the symptoms are a response of the pulp and periradicular tissues.

While there are no pathognomonic symptoms for identifying cracked teeth, some symptoms are more common than others. Anecdotally, pain on biting has been considered to be consistently present in cracked teeth.^12^ Extensive practice‐based network studies have, however, shown cold sensitivity is the predominant symptom.^13^ Interestingly, in a recent survey of Australian dentists, only 0.6% of respondents considered sensitivity to cold as a possible standalone symptom of cracked teeth.^12^ The difficulty in diagnosing cracked teeth is compounded as cold sensitivity could be due to other factors such as dentine hypersensitivity, tooth surface loss, caries, or microleakage or parafunction.^13^, ^14^ The variability in the presentation of cracked teeth to general dental practices compared with their presentation to referral‐based specialist practices has not received enough attention. For instance, in general practice, based on the series of papers published by the national dental practice‐based research network (NDPBRN), the majority of the cracked teeth were asymptomatic (55%). On the contrary, the more severe and complex, often with a contradicting combination of symptoms, are likely to be referred to specialist practices and tertiary referral centres.^9^, ^10^, ^15^

Determining whether a tooth is cracked or not, particularly in a restored tooth, is difficult.^12^ While the NDPBRN crack study resulted in a series of comprehensive papers on cracked teeth, they included only teeth where cracks were visible externally, potentially missing some cracked teeth.^13^ It is likely that the presence of cracked teeth is underreported.

The aim of this retrospective practice‐based cross‐sectional study was to:

- Identify the patient‐specific and tooth‐specific characteristics of individuals who presented to a private general dental practice and explore the relationship between crack presence and symptoms.
- Identify the predictors of cold sensitivity, positive bite test and certain crack characteristics.

## MATERIALS AND METHODS

**Fig. 1:**
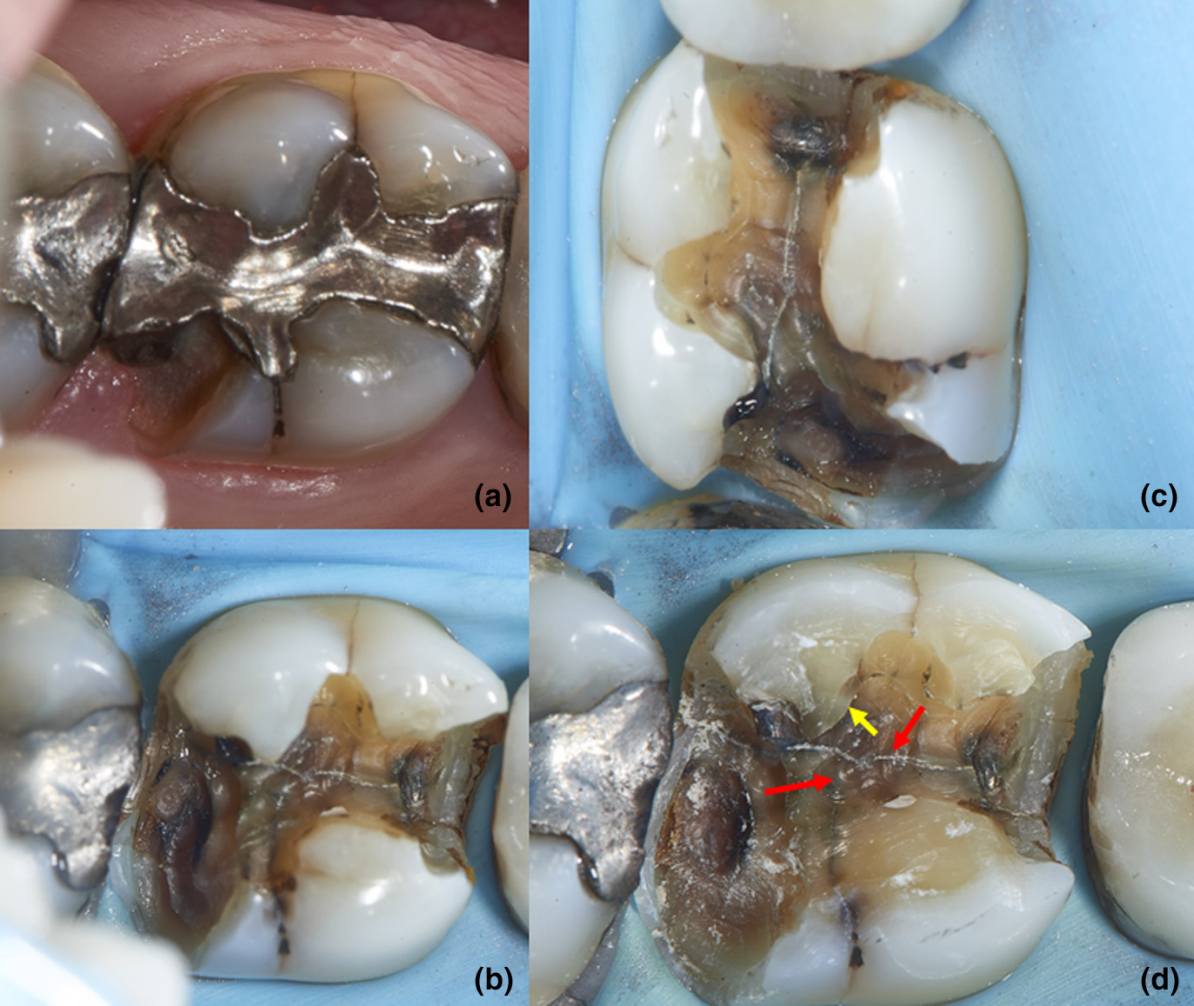
An example of a case of body and cuspal crack (a) through various stages of restoration removal, assessment and refinement (b–d). The mesiodistal and buccolingual (palatal) cracks are relatively easy to detect as they are on the pulp floor. Note the presence of intersecting cracks (red arrow) and that the cuspal cracks are not easily discernible in (b) and (c). The base of the cusps had to be specifically examined to pick up the cuspal crack (yellow arrow) on the mesiobuccal cusp (d).

This study was approved by the ethics review board of the University of Queensland, project number 2022/HE000285. In this study, a cracked tooth was defined as a thin surface disruption of enamel and dentin, and possibly cementum, of unknown depth or extension.^16^ Cracks in teeth were further divided according to their position in the tooth, with those that ran under a cusp recorded as a cuspal crack, and those that ran along the middle of the tooth (either buccolingually or mesiodistally) were recorded as a body crack (Fig. 1). Those cracks which joined to each other were called intersecting cracks. These categorisations were often made after removal of restoration and examination of the pulp floor of the cavity preparation and specific examination of the base of the cusps (Fig. 1).

A convenience sample was derived from consecutive patients' records belonging to the period 2014–2017 who visited a general dental practice in xxx, Australia, and who were diagnosed with cracks in teeth. All patients were examined by the same practitioner (PR) with Carl Zeiss Eyemag loupes 5.5X with Eyemag light. As a standard practice, preoperative and intraoperative photographs were taken at different angles at various stages of removing the restoration using a Nikon D850 DSLR camera (Nikon^®^, Japan) with a Nikon 105 macro lens and a 20 mm extension tube with a Metz Mecablitz 15 MS‐1 macro ring light (Metz‐ Werke GmbH&Co, Germany). The RAW images were post‐processed using Capture One (Capture One^®^, Denmark). The pulp sensibility tests were done using Frost Bite (ADM, Australia) sprayed on a number 2 cotton pellet and placed on the mid‐buccal surface of the tooth. The bite test was done by systematically asking the patient to bite on a Tooth Slooth^®^ while loading individual cusps, and pain on release was noted as a positive test. The clinical records were screened for mentions of cracks, and the associated sequence of clinical photographs was evaluated for specific tooth and crack characteristics (Fig. 1).

There were three inclusion criteria.

- Cases with a preoperative identification of a crack in a tooth.
- Cases where a crack was noticed only after the existing restoration was removed.
- Cases with a normal response to pulp sensibility testings and those with symptoms suggestive of reversible pulpitis.

The exclusion criteria were endodontically treated teeth, split teeth, fractured cusps, vertical root fractures, anterior teeth, external cracks, which were found to have no extension into dentin after restoration removal, and those teeth cracked as a result of traumatic dental injuries. Those with necrotic pulp or symptomatic irreversible pulpitis and periradicular pathosis were excluded.

A total of 152 patient records were obtained and analysed for completeness in terms of patient‐specific (age, gender, symptoms, periodontal status), tooth‐specific (occlusion, lateral excursive guidance, wear, tooth type and jaw, type of restoration, crack visibility before dismantling, pulp sensibility test, bite test and opposing teeth) and crack‐specific characteristics (direction of crack, number of cracks, location and staining). Five were excluded due to incomplete records, leaving 147 patient records for final analysis.

### Data analysis

Descriptive statistics were obtained from the Jamovi statistical program on patient‐, tooth‐ and crack‐specific characteristics. Further analysis of the data was done using a chi‐square test to explore the association of several independent variables (patient, tooth and crack characteristics) with symptom status (asymptomatic vs symptomatic).

Four other analyses followed this, and a *P*‐value of <0.05 was considered statistically significant.

- The objective of the first analysis was to assess the impact of patient, tooth and crack characteristics on developing cold sensitivity. A stepwise binomial logistic regression was performed to reduce multicollinearity and identify the best subset of predictors. Several iterations of the model building were performed, including variables with *P* value <0.20 and eliminating variables with *P* values >0.10 in subsequent iterations (unless clinically relevant). Akaike information criterion (AIC) was used to estimate how well the model fits the generated data. The initial model had an AIC of 149 and *R* ^2^ of 0.25, and the refined one had an AIC of 137 and *R* ^2^ (McFadden's) of 0.20. Multicollinearity was assessed using variance inflation factor (VIF), and the final model had a VIF in the range of 1.06–1.25, showing low multicollinearity.
- The objective of the second analysis was to assess the impact of patient, tooth and crack characteristics on yielding a positive bite test. The process was similar in terms of model building and optimisation. The initial model had an AIC of 167, *R* ^2^ (McFadden) of 0.34 and the VIF of individual predictors that ranged from 1.07 to 1.38. The refined model had an AIC of 151, *R* ^2^ (McFadden's) of 0.30 and VIF that ranged from 1.04 to 1.08.
- The objective of the third analysis was to assess the patient and tooth factors that could predict two crack characteristics. These characteristics were the number of cracks (multiple vs single) and crack type (intersecting vs single). A stepwise multinomial logistic regression model was built as explained earlier. The initial model had an AIC of 317, *R* ^2^ (Mc Faddens's) of 0.10 and VIF range of 1.09–1.35. The refined model had an AIC of 308, *R* ^2^ (McFadden's) of 0.07 and VIF range of 1.04–1.08.
- The final analysis was to assess the patient and tooth factors that could predict the presence of body and cuspal cracks. A stepwise binomial logistic regression model was built and optimised as mentioned earlier. The initial model had an AIC of 165, *R* ^2^ (Mc Fadden's) of 0.11 and VIF ranging from 1.11 to 1.44. The refined model had an AIC of 158, *R* ^2^ (McFadden's) of 0.07 and VIF range of 1.03–1.18.

## RESULTS

### Patient‐specific characteristics

The age, gender, type of symptoms, occlusion and periodontal status were explored to describe the baseline characteristics of patients. Mean age of the cohort was 62.3 (±10) years, with 51.7% above the age of 60 years. There were more females 57.1% than males 42.9%.

Interestingly, slightly over half of the patients who presented with cracked teeth were asymptomatic (55.1%). Those with symptoms had a singular or combination of symptoms such as thermal sensitivity (18.4%), pain on biting (15%), and combined symptoms of thermal sensitivity and pain on biting (11.6%). When all the symptomatic patients were clubbed together, thermal sensitivity was present in 40% (27/66), pain on biting in 33% (22/66) and combined symptoms in 25% of the symptomatic patients (17/66).

### Tooth‐specific characteristics

The most common tooth with an identified crack was the mandibular second molar (23.8%), and the least affected was the maxillary third molar (0.7%). The spread between molars and premolars was 80.3% and 19.7%, respectively, and between maxillary and mandibular teeth was 47.6% and 52.4%, respectively.

An amalgam restoration was present in 69.4% of the sample, of which single surface occlusal amalgam restorations were the most common (22.4%). Composite restorations accounted for 23.8% of the teeth, the most common composite restoration being three surface composite restorations (10.2%).

The majority of cracks (55.1%) were not visible before removing the restoration compared to 44.9% that were clinically visible. The response to the cold test was normal in 94.6%, whereas in 5.4%, it lingered for 10 s. The bite test was negative in 51.7%. The remaining 48.3% yielded a positive test.

Composite resin restorations were present in 36.1% of the opposing tooth, followed by amalgam restorations (32.7%), with the remaining belonging to various other categories mentioned in File S1.

### Crack‐specific characteristics

Specifically, four characteristics of the cracks were assessed; the direction, number of cracks, location and whether the crack was stained or not. Cracks running in the mesiodistal direction were seen in 48.3% of cracked teeth, whereas buccolingual cracks were only present in 1.4%. However, the common presentation was cracks running in both mesiodistal and buccolingual directions (50.3%). Most of the cracks were single cracks (44.2%). When multiple cracks were identified, some of these were found to intersect with each other in 27.9%. With regard to the location of cracks, most cracks were in the body of the tooth (72.8%) with standalone incomplete cusp cracks accounting for only 3.4% of the teeth. However, cracks in both the body and underneath the cusps were noticed in 23.8% of the teeth. Most of the cracks were not stained (53.7%) compared to 46.3% that were stained.

### Patient, tooth and crack characteristics and their relationship with symptomatic teeth (Table 1)

**Table 1:** Patient, tooth and crack characteristics that were significantly associated with symptom status (chi‐square test)

Factors	Counts n (%)	Symptom status		*P* value
		Asymptomatic n (row %)	Symptomatic n (row %)	
Age				0.04*
Less than 60 years	63 (42.9%)	30 (47.6%)	33 (52.4%)	
More than 60 years	84 (51.7%)	54 (64.3%)	30 (35.7%)	
Crack visible before removing restoration				<0.001
Yes	66 (44.9%)	28 (42.4%)	38 (57.6%)	
No	81 (55.1%)	56 (69.1%)	25 (30.9%)	
Staining				0.04
No stain	79 (53.7%)	39 (49.4%)	40 (50.6%)	
Stained	68 (46.3%)	45 (66.2%)	23 (33.8%)	
Location of crack				0.01
Body or cusp	112 (76.2%)	70 (62.5%)	42 (37.5%)	
Body & cusp	35 (23.8%)	14 (40.0%)	21 (60.0%)	

*Note*: * Statistically significant.

Chi‐square test revealed that symptomatic teeth were statistically significantly associated with patients who were 60 years and younger, cracks visible before restoration removal, unstained cracks and those cracks involving both the body and cusp of teeth.

### Factors associated with cold sensitivity (Table 2)

**Table 2:** Association of patient, tooth and crack characteristics with cold sensitivity (binomial logistic regression)

Predictors	*P* value	Odds ratio	Lower	Upper
95% confidence interval				
Marginal ridge (unrestored vs restored)	**0.03**	2.89	1.06	7.82
Crack visible before dismantling (yes‐no)	**0.02**	3.04	1.14	8.12
Status of antagonist tooth (unrestored vs restored)	0.09	2.31	0.87	6.12
Staining of crack (stained vs non‐stained)	**0.01**	0.30	0.11	0.74
Body & cusp cracks (yes‐no)	**0.02**	3.11	1.13	8.54

*Note*: Bold values were statistically significant.

Binomial logistic regression revealed that three variables were positively associated with cold sensitivity. Cracked teeth with unrestored marginal ridges (OR 2.89), cracks visible before restoration removal (OR 3.04) and cracks involving both the body and cusps (OR 3.11) were more likely to be associated with cold sensitivity. The presence of stained cracks was negatively associated with cold sensitivity (OR 0.30).

### Factors associated with positive bite test (Table 3)

**Table 3:** Association of patient, tooth and crack characteristics with positive bite test (binomial logistic regression)

Predictors	*P*‐value	Odds ratio	Lower	Upper
95% confidence interval				
Tooth (molar vs premolar)	**0.02**	3.71	1.16	11.78
Pain on biting (presence vs absence)	**<0.001**	36.96	7.79	175.34
Crack visible before restoration removal (Yes vs No)	**0.00**	3.44	1.49	7.92
Number of cracks (multiple vs single)	0.06	2.24	0.95	5.30

*Note*: Bold values were statistically significant.

The key finding was that not all cracked teeth yield a positive bite test, with nearly half of the sample (51.7%) having a negative bite test. Among the positive bite tests, 36.1% were symptomatic and 12.2% were asymptomatic. Binomial logistic regression revealed that three variables were associated with a positive bite test. Molar teeth (OR 3.71), those presenting with a primary symptom of pain on biting (OR 36.96) and those with cracks visible before restoration removal (OR3.44), were more likely to yield a positive bite test.

### Factors associated with number of cracks (Table 4)

**Table 4:** Association of patient, tooth characteristics with number and type of cracks (multinomial logistic regression)

Crack characteristics: number of cracks	Predictor	*P* value	Odds ratio	Lower	Upper
				95% Confidence interval	
Multiple‐single	Tooth (molar vs premolar)	0.38	1.51	0.58	3.92
	Jaw type (mandibular vs maxillary)	0.04	0.40	0.16	0.95
	Type of restoration (amalgam vs others)	0.08	2.17	0.89	5.25
Intersecting‐single	Tooth (molar vs premolar)	0.01	8.79	1.83	42.10
	Jaw type (mandibular vs maxillary)	0.15	0.52	0.21	1.28
	Type of restoration (amalgam vs others)	0.00	4.81	1.68	13.78

Multinomial logistic regression revealed that those teeth with amalgam restoration were more likely to have multiple cracks (OR2.17) while mandibular teeth were negatively associated with multiple cracks (OR 0.40) compared with maxillary teeth. Intersecting cracks were positively associated with molars (OR 8.79) and those teeth with amalgam restorations (OR 4.81).

### Factors associated with the location of cracks (Table 5)

**Table 5:** Association of patient, tooth characteristics with combined body & cusp cracks (binomial logistic regression)

Crack characteristics: location	Predictor	*P* value	Odds ratio	Lower	Upper
				95% Confidence interval	
Body & cusp crack (presence vs absence)	Marginal ridge (restored vs intact)	**0.02**	3.22	1.18	8.77
	Type of restoration (amalgam vs others)	0.20	1.85	0.71	4.82
	Crack visible before restoration removal (yes vs no)	**0.01**	3.27	1.40	7.63

*Note*: Bold values were statistically significant.

The model building iterations could not identify any subset of variables that could predict body versus cusp cracks. However, there were two variables that could predict teeth with body and cusp cracks. Teeth with restored marginal ridges (OR 3.22) and cracks visible before removal of restoration (OR 3.27) were more likely to be associated with body and cusp cracks.

## DISCUSSION

This study evaluated the characteristics of patients with cracked teeth at three different levels, namely, patient‐specific, tooth‐specific and crack‐specific characteristics, and explored their relationship. There are some interesting similarities and differences between the current study and that of NDPBRN crack studies. Both reported that around 55% of the patients were asymptomatic. One of the key findings is that both NDPBRN studies and this study demonstrate that most of the patients with cracked teeth presenting to general dental practice are asymptomatic compared to those reported in specialist endodontic practice.^2^, ^9^, ^10^, ^15^ Among the symptomatic patients, thermal sensitivity (particularly sensitivity to cold) was the predominant symptom in both NDPBRN studies and this study, followed by pain on biting. There was a slight difference in the prevalence of the combination of symptoms, with the current study reporting 25%, whereas the NDPBRN reported 35%.^13^

### Key factors associated with symptoms

#### Age

The current study showed symptoms generally, and cold sensitivity in particular, were clearly more likely present in individuals less than 60 years. The relative reduction of cold sensitivity with increasing age (above 60) may be due to multiple factors. Ageing results in stiffening of the collagen matrices and affects the apatite density and distribution.^17^ This results in a reduction of the crack initiation toughness and maximum crack growth resistance values by about 5% per decade.^18^ Thus, the increased rate of crack initiation and propagation in dentine with age allows limited time for persistent odontoblast stimulation^19^ and potentially culminates in acute toothache rather than cold sensitivity. By contrast, younger dentine allows inelastic deformation of the mineralised collagen matrix, microcracking of the peritubular dentine and formation of unbroken ligaments behind the advancing crack^18^, thereby slowing the process and allowing more time, often resulting in cold sensitivity. Further, in those above 60, there is significant reduction in the number of odontoblasts and evidence of odontoblast apoptosis^20^ possibly resulting in lesser symptoms.

#### Cracks visible before restoration removal

Even though NDPBRN crack studies had close to 3000 teeth, they were all with at least one externally visible crack.^13^, ^21^ A key differentiating factor of the current study was that in contrast to the NDPBRN crack studies^13^, ^21^ most (55.1%) of the cracks in the current study were only visible following removal of restoration. In addition, cracks not visible before restoration removal were usually asymptomatic. This highlights the need for diligent examination of the remaining tooth structure following routine restoration replacements.

In treating teeth where cracks are visible before removal of restoration, clinicians should specifically look for cracks at the cuspal base, as teeth with visible cracking are more likely to have both body and cuspal cracks. This was another key contribution of this study.

#### Staining

Similar to the NDPBRN crack study (13), stained cracks were inversely associated with symptoms. This negative association was also confirmed with cold sensitivity. The reason for the inverse relation is likely due to stained cracks being stable and perhaps long‐standing, resulting in reactionary dentine formation and dentine tubule sclerosis.

#### Marginal ridge

**Fig. 2:**
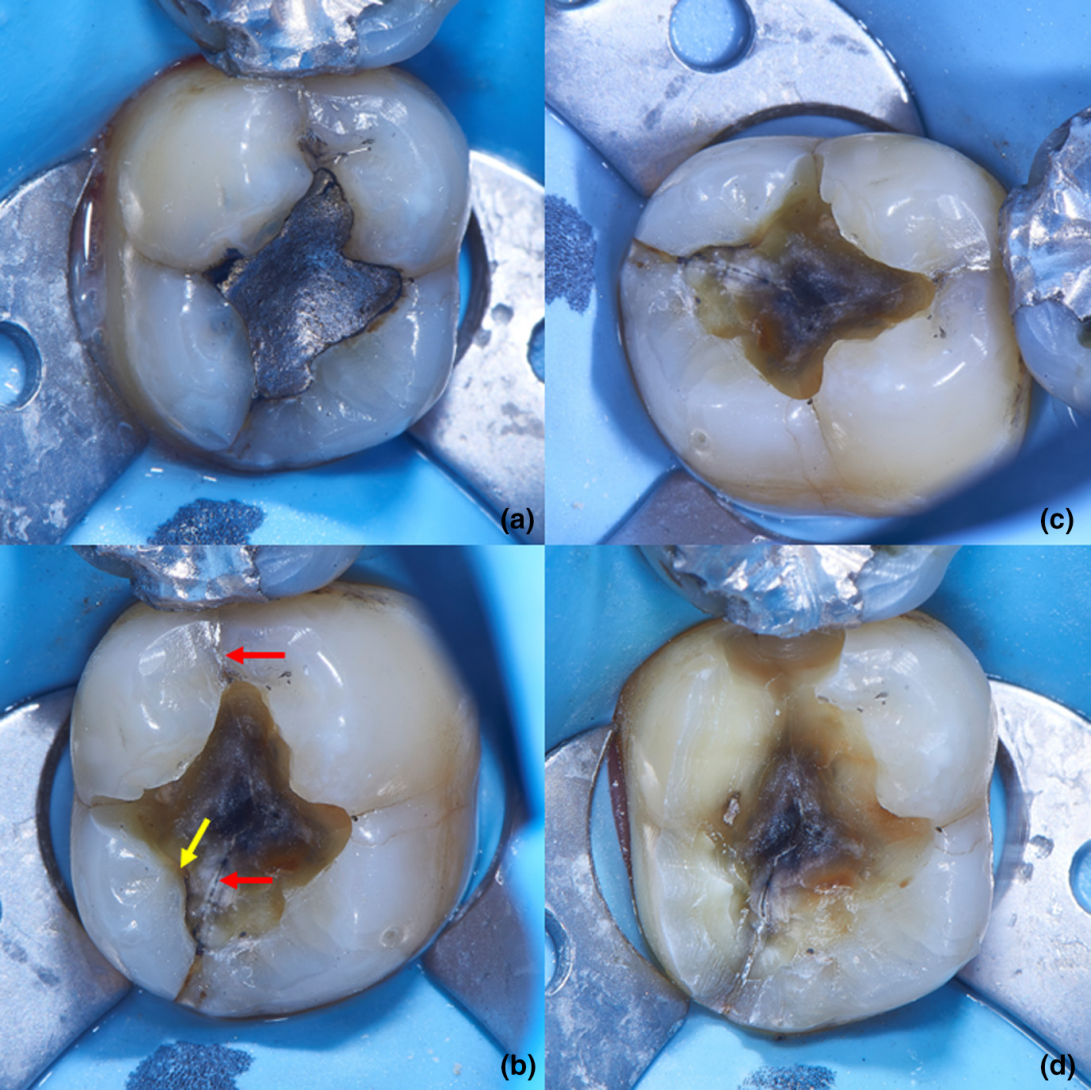
An example of a case where a crack was visible before removal of restoration (a). The marginal ridges were unrestored and cracked. (b and c) Removal of amalgam restoration and examination of the pulp floor revealed body cracks (red arrow). Specific examination of the individual cuspal base revealed cuspal crack (yellow arrow) involving distolingual and mesiolingual cusps. (d) Completion of preparation and reduction of distolingual and mesiolingual cups.

The current study showed a higher odds ratio (OR: 2.89) of cold sensitivity in cracked teeth with unrestored marginal ridges. It is likely that though the marginal ridges were unrestored, they were cracked (Fig. 2). The relation between loss of integrity of marginal ridge and increased cuspal deflection is well documented.^22^ In addition, continuous occlusal loading similar to that in parafunction results in the deflected cusp requiring more recovery time to return to its original position.^23^ Increased cuspal deflection along with the presence of cracks may have been the cause of cold sensitivity.

The presence of cracks, along with prolonged and repeated cuspal flexion, may facilitate odontoblasts to act as mechanosensory receptors expressing thermosensitive transient receptor potential ion channels (thermo‐TRP) resulting in hyperalgesia and temperature sensitivity.^24^ Other factors involve fluid movement in dentinal tubules, resulting in cellular perturbation of odontoblasts and neurophysiological changes to prolonged noxious stimuli, causing sensitisation of A delta fibres and upregulation of sodium channels to prolonged noxious stimuli.^25^

#### Body and cusp cracks

The strong association of cold sensitivity and positive bite test with body and cuspal cracks suggests that clinicians should look for cracks in these locations when these combinations of symptoms are present. This combination of symptoms could possibly be explained using two key factors: severe cuspal deflection and algoneurons.

Panitvisai and Messer in their classic study on cuspal deflection on mandibular molars compared the effect of mesio‐occlusal preparation with cuspal isolation.^22^ Cuspal isolation was created by placing a vertical groove buccal‐lingually just distal to the mesial cusps, extending to the cementoenamel junction and completely isolating the mesial cusps from the remainder of the crown. Loading such isolated cusps created the maximum deflection compared with all the other types of conservative and extensive preparations used in their study. Though their model of cuspal isolation was an extreme scenario, body and cuspal cracks explored in the current study can be considered as a type of cuspal isolation and possibly result in significant cuspal deflection.

Secondly, unlike traditional nociceptors, the A delta fibre low threshold mechanoreceptors (LTMs) such as algoneurons in the dentine pulp complex can be stimulated by activities such as air puffs, light pressure, vibration and tactile sensations.^26^ It is possible that body and cuspal cracks provide enough mechanical instability for stimulation of these algoneurons.^26^

### Key factors associated with crack number and location

Diagnostic tools used for crack teeth are poorly studied against gold standards, and their sensitivity and specificity are largely unknown.^27^ These tools may provide some information on the presence or absence of cracks and limited insight into 3‐dimensional mapping of crack anatomy. This study has shown that molars are associated with intersecting cracks compared with single cracks. This is likely due to the occlusal morphology of molars, such as deep occlusal fissures and buccal and lingual grooves providing the surface defects for crack initiation.^28^ The restorative implications of intersecting cracks are unknown, but the impact on the structure's integrity seems intuitive.

With regard to the crack location, two positive associations were identified. A restored marginal ridge was associated with body and cuspal cracks. This is not a surprising finding as demonstrated by Panitvisai and Messer.^22^ Interestingly, cracks visible before restoration removal were associated with body and cuspal cracks. These are easy to miss when they exist in combination and call for careful evaluation of not just the cavity floor but also the base of the cusps after restoration removal.

This cross‐sectional practice‐based study has identified key factors associated with symptomatic cracks, sensitivity to cold and positive bite tests. It has also identified factors relevant to crack number and location. However, as this study was cross‐sectional without a control group, the associations drawn have limited external validity. The readers need to realise that the results reported in this study are predominantly characteristics of cracks associated with restorations from a convenience sample and unrestored teeth accounted for only 6.8% of the sample. This critical differentiation between cracks in unrestored teeth and restoration‐associated cracks explains some of the differences in symptoms reported across the literature. Further, the samples under unrestored teeth included both intact and unrestored carious teeth. This clustering was done due to low number if they were sub‐categorised. Another limitation is that this study did not use CO_2_ ice for pulp testing, nor was transillumination used to detect cracks. However, unlike the NDPBRN crack study teeth (Hilton *et al*., 2018), an external crack was not an inclusion criterion, and we have captured a more realistic sample relevant to general dental practice.

## CONCLUSION

In general dental practice, there is a wide range of presentations of cracked teeth, from being mostly asymptomatic to those with cold sensitivity, pain on biting, a combination of symptoms and acute toothache. Following routine restoration replacements, particularly in asymptomatic teeth, the integrity of the remaining tooth structure should be comprehensively evaluated to rule out the presence of cracks. The authors also hope to get the reader's attention to the difference in the characteristics of cracked teeth seen routinely in general practice to those reported in the literature from specialist practices.

## AUTHOR CONTRIBUTIONS

**P Renner:** Conceptualization; investigation; writing – review and editing; methodology; resources; data curation; project administration. **U Krishnan:** Conceptualization; investigation; writing – original draft; writing – review and editing; software; formal analysis; methodology; data curation; project administration; validation; visualization. **A Moule:** Conceptualization; methodology; writing – review and editing; project administration; supervision. **M Swain:** Conceptualization; methodology; writing – review and editing; project administration; supervision.

## Supporting information


Data S1.
