# Supplementary material for: Characteristics of cracks in posterior teeth and factors associated with symptoms: a cross‐sectional practice‐based observational study
Source: Aust Dent J. 2025 Apr 24;70(3):181–9. doi: 10.1111/adj.13075 (PMC12368304; doi:10.1111/adj.13075)
Supplement: Supplementary file 1 — Data S1. [file ADJ-70-181-s001.docx]

**Supplementary file 1: Distribution of cracked teeth according to patient, tooth and crack characteristics and their relationship with symptom status**

| **Factors** | **Counts**  **n (%)** | **Symptom status** | | **P value** |
| --- | --- | --- | --- | --- |
|  | | **Asymptomatic**  **n (row %)** | **Symptomatic**  **n (row %)** |  |
| **Age**  Less than 60 years |  |  |  | **0.04^*^** |
|  | 63 (42.9%) | 30 (47.6%) | 33 (52.4%) |  |
| More than 60 years | 84 (51.7%) | 54 (64.3%) | 30 (35.7%) |  |
| **Gender** |  |  |  | 0.31 |
| Male | 63 (42.9%) | 39 (61.9%) | 24 (38.1%) |  |
| Female | 84 (57.1%) | 45 (53.6%) | 39 (46.4%) |  |
| **Tooth type** |  |  |  | 0.05 |
| Max first PM | 17 (11.6%) | 11 (64.7%) | 6 (35.3%) |  |
| Max second PM | 4 (2.7%) | 2 (50.0%) | 2 (50.0%) |  |
| Max First Molar | 29 (19.7%) | 18 (62.1%) | 11 (37.9%) |  |
| Max second Molar | 19 (12.9%) | 6 (31.6%) | 13 (68.4%) |  |
| Mand first PM | 2 (1.4%) | 2 (100.0%) | 0 (0.0%) |  |
| Mand second PM | 7 (4.8%) | 6 (85.7%) | 1 (14.3%) |  |
| Mand first Molar | 27 (18.4%) | 20 (74.1%) | 7 (25.9%) |  |
| Mand second Molar | 35 (23.8%) | 17 (48.6%) | 18 (51.4%) |  |
| Mand third molar | 6 (4.1%) | 2 (33.3%) | 4 (66.7%) |  |
| Max third Molar | 1 (0.7%) | 0 (0.0%) | 1 (100.0%) |  |
| **Jaw type** |  |  |  | 0.31 |
| Maxillary | 70 (47.6%) | 37 (52.9%) | 33 (47.1%) |  |
| Mandibular | 77 (52.4%) | 47 (61.0%) | 30 (39.0%) |  |
| **Restorative status of tooth** |  |  |  | 0.07 |
| Oc Am | 33 (22.4%) | 16 (48.5%) | 17 (51.5%) |  |
| MO/BO Am | 12 (8.2%) | 4 (33.3%) | 8 (66.7%) |  |
| DO Am | 15 (10.2%) | 14 (93.3%) | 1 (6.7%) |  |
| MOD/MOL/DOL/MOB Am | 29 (19.7%) | 15 (51.7%) | 14(48.3%) |  |
| MODB Am | 3 (2.0%) | 2 (66.7%) | 1 (33.3%) |  |
| MODL/DOBL Am | 8 (5.4%) | 6 (75.0%) | 2 (25.0%) |  |
| MODBL Am | 2 (1.4%) | 1 (50.0%) | 1 (50.0%) |  |
| Oc CR | 6 (4.1%) | 3 (50.0%) | 3 (50.0%) |  |
| MO/LO/BO CR | 9 (6.1%) | 5 (55.6%) | 4 (44.4%) |  |
| DO CR | 1 (0.7%) | 0 (0.0%) | 1 (100.0%) |  |
| MOD/MOL/DOL CR | 15 (10.2%) | 10 (66.7%) | 5 (33.3%) |  |
| MODL CR | 1 (0.7%) | 0 (0.0%) | 1 (100.0%) |  |
| MODB CR | 4 (2.7%) | 4 (100.0%) | 0 (0.0%) |  |
| MODBL CR | 4 (2.7%) | 3 (75.0%) | 1 (25.0%) |  |
| Caries | 1 (0.7%) | 0 (0.0%) | 1 (100.0%) |  |
| Unrestored | 4 (2.7%) | 1 (25.0%) | 3 (75.0%) |  |
| **Status of marginal ridge** |  |  |  | 0.06 |
| Intact Marginal ridge | 44 (29.9%) | 20 (45.5%) | 24 (54.5%) |  |
| Lost marginal ridge | 103 (70.1%) | 64 (62.1%) | 39 (37.9%) |  |
| **Crack visible before dismantling** |  |  |  | **<0.001** |
| Yes | 66 (44.9%) | 28 (42.4%) | 38 (57.6%) |  |
| No | 81 (55.1%) | 56 (69.1%) | 25 (30.9%) |  |
| **Probing depth** |  |  |  | 0.42 |
| </=3mm | 138 (93.9%) | 80 (58.0%) | 58 (42.0%) |  |
| >3mm | 9 (6.1%) | 4 (44.4%) | 5 (55.6%) |  |
| **Generalised periodontitis** |  |  |  | 0.46 |
| Nil | 143 (97.3%) | 81 (56.6%) | 62 (43.4%) |  |
| More than 1Q with>3mm probing | 4 (2.7%) | 3 (75.0%) | 1 (25.0%) |  |
| **Status of opposing tooth** |  |  |  | 0.31 |
| Oc Am | 22 (15.0%) | 9 (40.9%) | 13 (59.1%) |  |
| MO/BO Am | 6 (4.1 %) | 4 (66.7%) | 2 (33.3%) |  |
| DO Am | 5 (3.4%) | 4 (80.0%) | 1 (20.0%) |  |
| MOD/MOL/DOL/MOB Am | 8 (5.4%) | 5 (62.5%) | 3 (37.5%) |  |
| MODB Am | 1 (0.7%) | 1 (100.0%) | 0 (0.0%) |  |
| MODL/DOBL Am | 3 (2.0%) | 3 (100.0%) | 0 (0.0%) |  |
| MODBL Am | 4 (2.7%) | 3 (75.0%) | 1 (25.0%) |  |
| Oc CR | 9 (6.1%) | 4 (44.4%) | 5 (55.6%) |  |
| MO/LO/BO CR | 10 (6.8%) | 4 (40.0%) | 6 (60.0%) |  |
| DO CR | 4 (2.7%) | 3 (75.0%) | 1 (25.0%) |  |
| MOD/MOL/DOL CR | 18 (12.2%) | 13 (72.2%) | 5 (27.8%) |  |
| MODL CR | 8 (5.4%) | 6 (75.0%) | 2 (25.0%) |  |
| MODB CR | 2 (1.4%) | 1 (50.0%) | 1 (50.0%) |  |
| MODBL CR | 2 (1.4%) | 1 (50.0%) | 1 (50.0%) |  |
| Unrestored | 35 (23.8%) | 14 (40.0%) | 21 (60.0%) |  |
| Zirc Crown | 5 (3.4%) | 4 (80.0%) | 1 (20.0%) |  |
| Partial Denture | 2 (1.4%) | 2 (100.0%) | 0 (0.0%) |  |
| Unopposed | 1 (0.7%) | 1 (100.0%) | 0 (0.0%) |  |
| Porcelain Crown | 1 (0.7%) | 1 (100.0%) | 0 (0.0%) |  |
| Gold Crown | 1 (0.7%) | 1 (100.0%) | 0 (0.0%) |  |
| **Wear** |  |  |  | 0.97 |
| No wear | 24 (16.3%) | 14 (58.3%) | 10 (41.7%) |  |
| Slight enamel wear | 42 (28.6%) | 23 (54.8%) | 19(45.2%) |  |
| Moderate dentin wear without flattened cusp | 68 (46.3%) | 40 (58.8%) | 28 (41.2%) |  |
| Heavy wear with flattened cusp | 13 (8.8%) | 7 (53.8%) | 6 (46.2%) |  |
| **Type of guidance** |  |  |  | 0.47 |
| Canine guidance | 82 (55.8%) | 49 (59.8%) | 33 (40.2%) |  |
| Group function | 65 (44.2%) | 35 (53.8%) | 30 (46.2%) |  |
| **Occlusion** |  |  |  | 0.40 |
| Class 1 Molar relation | 136 (92.5%) | 77 (56.6%) | 59 (43.4%) |  |
| Posterior Cross bite | 8 (5.4%) | 5 (62.5%) | 3 (37.5%) |  |
| Edge- Edge | 2 (1.4%) | 2 (100.0%) | 0 (0.0%) |  |
| Class 3 | 1 (0.7%) | 0 (0.0%) | 1 (100.0%) |  |
| **Crack direction** |  |  |  | 0.71 |
| Mesio distal | 71 (48.3%) | 43 (60.6%) | 28 (39.4%) |  |
| Bucco lingual | 2 (1.4%) | 1 (50.0%) | 1 (50.0%) |  |
| Both | 74 (50.3%) | 40 (54.1%) | 34 (45.9%) |  |
| **Number of cracks** |  |  |  | 0.33 |
| Single | 65 (44.2%) | 40 (61.5%) | 25 (38.5%) |  |
| Multiple/Intersecting | 82 (55.8%) | 44 (53.7%) | 38 (46.3%) |  |
| **Stainin**g |  |  |  | **0.04** |
| No stain | 79 (53.7%) | 39 (49.4%) | 40 (50.6%) |  |
| Stained | 68 (46.3%) | 45 (66.2%) | 23 (33.8%) |  |
| **Location of crack** |  |  |  | **0.01** |
| Body or Cusp | 112 (76.2%) | 70 (62.5%) | 42 (37.5%) |  |
| Body & cusp | 35 (23.8%) | 14 (40.0%) | 21 (60.0%) |  |
